# A multilevel pan-cancer map links gene mutations to cancer hallmarks

**DOI:** 10.1186/s40880-015-0050-6

**Published:** 2015-09-14

**Authors:** Theo A. Knijnenburg, Tycho Bismeijer, Lodewyk F. A. Wessels, Ilya Shmulevich

**Affiliations:** Institute for Systems Biology, Seattle, WA 98109 USA; Bioinformatics and Statistics, Division of Molecular Carcinogenesis, Netherlands Cancer Institute, 1066 CX Amsterdam, The Netherlands

**Keywords:** Cancer systems biology, Cancer hallmarks, Gene mutations, Multilevel model

## Abstract

**Background:**

A central challenge in cancer research is to create models that bridge the gap between the molecular level on which interventions can be designed and the cellular and tissue levels on which the disease phenotypes are manifested. This study was undertaken to construct such a model from functional annotations and explore its use when integrated with large-scale cancer genomics data.

**Methods:**

We created a map that connects genes to cancer hallmarks via signaling pathways. We projected gene mutation and focal copy number data from various cancer types onto this map. We performed statistical analyses to uncover mutually exclusive and co-occurring oncogenic aberrations within this topology.

**Results:**

Our analysis showed that although the genetic fingerprint of tumor types could be very different, there were less variations at the level of hallmarks, consistent with the idea that different genetic alterations have similar functional outcomes. Additionally, we showed how the multilevel map could help to clarify the role of infrequently mutated genes, and we demonstrated that mutually exclusive gene mutations were more prevalent in pathways, whereas many co-occurring gene mutations were associated with hallmark characteristics.

**Conclusions:**

Overlaying this map with gene mutation and focal copy number data from various cancer types makes it possible to investigate the similarities and differences between tumor samples systematically at the levels of not only genes but also pathways and hallmarks.

**Electronic supplementary material:**

The online version of this article (doi:10.1186/s40880-015-0050-6) contains supplementary material, which is available to authorized users.

## Background

A central challenge in cancer research is to create models that bridge the gap between the molecular level on which interventions can be designed and the cellular and tissue levels on which the disease phenotypes are manifested. This is a daunting task. Cancer genomics research in the last decade has revealed the enormous complexity of this disease. Essential to the cancer phenotype and to its understanding are interactions between genes, between signaling pathways, and between cells. The latter interaction is exemplified by the important role of tumor heterogeneity [1, 2] and the relationship between the tumor and its environment [3, 4].

The complexity of cancer is reflected by the notion that cancer should not be considered as one disease but as a set of many diseases. In addition to traditional characteristics, including body location and morphology, cancers are distinguished by differences in their (epi)genomic signatures, gene and protein expression levels, and hyperactivated or deactivated pathways. Importantly, these differences at the molecular level are expected to enable personalized treatment strategies [5–7].

However, all cancers share the same set of deregulated biological processes, termed the hallmarks of cancer [8, 9]. How can we understand that tumors that are very different at the molecular level are similar when observed at a higher level of functional abstraction? More importantly, can this mapping that integrates the molecular characteristics and the disease phenotype lead to new hypotheses about biological mechanisms and therapy?

We have attempted to address these questions by creating a map that connects genes via pathways to hallmarks. By projecting gene mutation data from various cancer types on this map, we investigated the similarities and differences between these cancer types at the levels of not only genes but also pathways and hallmarks.

We considered mutually exclusive (ME) and co-occurring (CO) genes in the context of the multilevel map. In several studies, it has been observed that gene mutations that affect a pathway tend to be altered in an ME pattern [10]. The rationale behind that observation is that once a gene involved in a pathway is mutated, a second mutation affecting that pathway does not confer a further selective advantage to the cancer cell. The large number of pathways in the multilevel map allowed us to systematically test whether there are indeed many ME mutations in the pathways. Interestingly, ME associations are typically expected within a pathway and not across pathways [11]. This begs the question of whether there are ME associations between pairs of genes that are not part of the same pathway but link to the same hallmark, or whether there are many CO associations at the level of hallmarks. A CO association, which is on the other end of the spectrum from an ME association, means that genes are frequently found mutated together across cancer samples. The deregulation of distinct biological functions by these CO mutations may be necessary to acquire certain hallmark characteristics. Finally, we employed the map to assess whether genes that are not significantly frequently mutated (SFM) in a cancer type, but are mutated in a small number of samples, have a role in enabling cancer hallmark characteristics. Recent cancer genome studies have clearly demonstrated the extensive mutational heterogeneity in cancers [12]; relatively few genes are SFM (and can be detected as such by statistical approaches), whereas most genes are mutated in a small number of samples. The functional role of these infrequently mutated genes is unclear. Here, we employed the multilevel map to elucidate the functional role of these genes.

## Methods

### Multilevel map

To link 1384 genes to 343 pathways and 10 hallmarks, we integrated information from the Pathway Interaction Database (PID) [13] and the Gene ontology (GO) [14]. The PID consists of 167 curated signaling pathways important in cancer. PID pathways involve multiple GO processes, which are the endpoints of signaling branches in the pathway. The 343 pathways in our topology comprise the genes within a PID pathway upstream of a GO process. These GO processes were linked to hallmarks by checking whether they are child processes of general GO categories that are representative of the cancer hallmarks. For example, in the PID “p53 pathway” there are 7 genes in a signaling cascade that regulate the GO “apoptotic process,” which is a child process of “programmed cell death,” which is linked to the hallmark “resisting cell death.” Table 1 lists the mapping from GO processes to hallmarks. These 10 cancer hallmarks consist of the 6 originally defined hallmarks [8] augmented by 2 emerging hallmarks and 2 enabling characteristics [9]. This mapping was performed by the authors with the help of domain experts at the Netherlands Cancer Institute. It is similar to a previous mapping [15]. Because multiple pathways can be extracted from one PID pathway, the pathways in the topology are labeled with the PID pathway name followed by an index. See “Multilevel map” in the Additional file 1 section for details.

**Table 1:** Mapping from hallmarks to Gene ontology (GO) processes

Hallmark	No. of genes	No. of pathways	Linked GO processes and function	
Sustaining proliferative signaling	343	50	GO:0008283, cell proliferation	
			GO:0016049, cell growth	
			GO:0007049, cell cycle	
			GO:0051301, cell division	
			GO:0008284, positive regulation of cell proliferation	
			GO:0030307, positive regulation of cell growth	
			GO:0045787, positive regulation of cell cycle	
			GO:0051781, positive regulation of cell division	
Evading growth suppressors	182	13	GO:0009968, negative regulation of signal transduction	
			GO:0008285, negative regulation of cell proliferation	
			GO:0030308, negative regulation of cell growth	
			GO:0045786, negative regulation of cell cycle	
			GO:0051782, negative regulation of cell division	
Resisting cell death	404	57	GO:0012501, programmed cell death	
			GO:0043067, regulation of programmed cell death	
Replicative immortality	2	1	GO:0090398, cellular senescence	
			GO:0032200, telomere organization	
			GO:0000723, telomere maintenance	
			GO:0032204, regulation of telomere maintenance	
			GO:0001302, replicative cell aging	
			GO:1900062, regulation of replicative cell aging	
			GO:2000772, regulation of cellular senescence	
Sustained angiogenesis	105	12	GO:0001525, angiogenesis	
			GO:0045765, regulation of angiogenesis	
			GO:0001570, vasculogenesis	
			GO:2001212, regulation of vasculogenesis	
			GO:0008015, blood circulation	
Tissue invasion and metastasis	614	82	GO:0007155, cell adhesion	
			GO:0001837, epithelial-to-mesenchymal transition	
			GO:0016477, cell migration	
			GO:0030155, regulation of cell adhesion	
			GO:0030030, cell projection organization	
			GO:0030036, actin cytoskeleton organization	
			GO:0030030, cell projection organization	
			GO:0034330, cell junction organization	
			GO:0042330, taxis	
			GO:0007163, establishment or maintenance of cell polarity	
Genome instability	124	7	GO:0006281, DNA repair	
			GO:0031570, DNA integrity checkpoint	
			GO:0045005, maintenance of fidelity involved in DNA-dependent DNA replication	
			GO:0006282, regulation of DNA repair	
Tumor-promoting inflammation	84	9	GO:0006954, inflammatory response	
			GO:0002367, cytokine production involved in immune response	
			GO:0002718, regulation of cytokine production involved in immune response	
			GO:0042060, wound healing	
			GO:0061041, regulation of wound healing	
			GO:0050727, regulation of inflammatory response	
			GO:0042533, tumor necrosis factor biosynthetic process	
Reprogramming energy metabolism	8	2	GO:0006006, glucose metabolic process	
			GO:0046323, glucose import	
			GO:0006096, glycolysis	
			GO:0071456, cellular response to hypoxia	
Evading immune destruction	150	19	GO:0006955, immune response	
			GO:0002418, immune response to tumor cells	
			GO:0002837, regulation of immune response to tumor cells	
			GO:0020012, evasion or tolerance of host immune response	
			GO:0006897, endocytosis	
Other	1047	142		

### Mutation data

Mutation data for 1384 genes and 2740 samples from 10 different cancer types were obtained from The Cancer Genome Atlas (TCGA) [12, 16]. The data were represented as a binary matrix, i.e., a gene is either aberrated or not. The 10 solid cancer types, including the TCGA abbreviations used in this work, are listed in Table 2. The binary mutation calls were derived from exome-sequencing data (all non-silent mutations) and copy number variation data (focal amplifications and deletions). See “Mutation data” and “Significantly mutated and altered copy number genes” in the Additional file 1 section for details.

**Table 2:** Ten tumor types and their abbreviations

Abbreviation	Description	No. of samples
BLCA	Bladder urothelial carcinoma	95
BRCA	Breast invasive carcinoma	637
CORE	Colon/rectum adenocarcinoma	219
GBM	Glioblastoma multiforme	268
HNSC	Head and neck squamous cell carcinoma	296
KIRC	Kidney renal clear cell carcinoma	338
LUAD	Lung adenocarcinoma	169
LUSC	Lung squamous cell carcinoma	177
OV	Ovarian serous cystadenocarcinoma	301
UCEC	Uterine corpus endometrioid carcinoma	240

### Projecting mutation data on the multilevel map

The gene mutation data of the 2740 TCGA tumor samples were projected onto the map. We followed a straightforward strategy to propagate these mutation calls from the level of genes to the levels of pathways and hallmarks. If a sample had a mutation in at least one gene within a pathway, the mutational investment (MI) score of the sample in that pathway was set to 1; otherwise, it was set to 0. In other words, we implemented a logical OR function when going from genes to pathways, where at least one of the inputs (mutation calls for genes in the pathway) should be 1 to get an output of 1 (pathway MI). Similarly, if a sample had a mutation in at least one gene that links to a hallmark, the MI score of the sample in that hallmark was set to 1, and otherwise, it was set to 0. For each tumor sample, MI scores are thus binary calls at the levels of pathways and hallmarks, and they indicate the potential deregulation of the pathway and the potential enabling of the cancer hallmark, respectively. See “Random map rewiring” in the Additional file 1 section for details.

### Mutual exclusivity and co-occurrence analysis

We employed a statistical analysis to detect ME and CO associations of pairs of genes across all cancer types. Within each cancer type, we determined the number of samples that have binary mutation calls for both members of a pair of genes. This “overlap” was assessed for ME associations, i.e., an overlap smaller than expected by chance, and for CO associations, i.e., an overlap larger than expected by chance. Gene pairs were grouped into three categories: (1) pairs of genes that were part of the same pathway for at least one of the pathways in the multilevel map, termed “pathway pairs;” (2) pairs of genes that were not part of the same pathway, yet impinged on the same hallmark for at least one of the hallmarks, termed “hallmark pairs;” and (3) pairs of genes that were neither part of the same pathway nor impinged on the same hallmark, termed “control pairs.” We tested all pairs of genes in which both genes had at least 25 mutations and either were part of the same pathway or were linked to the same hallmark. These analyses were performed for each cancer type separately. We used BiRewire [17] to create the appropriate null distribution for these tests. Specifically, for the binary mutation matrix of each cancer type, 10,000 permuted matrices were created. The observed overlap of mutated samples for a pair of genes in the original binary mutation matrix was compared with the overlap values derived from the 10,000 permuted matrices. Enhanced *P* value Estimation for Permutation Test (EPEPT) [18, 19] was used to compute *P* values for these permutation tests. Associations were called significant when *P* ≤ 1/*n*, where *n* was the total number of tests. If *n* was smaller than 20, the *P* value threshold was set to 0.05. This Bonferroni correction for multiple testing results in a per-family error rate of 1. ME and CO associations were tested separately. We did not test for CO for pairs of genes from the same chromosome to avoid spurious associations due to arm level copy number gain or loss.

### Tail strength

The overall amount of detectable ME and CO associations was measured by the tail strength (TS) statistic [20]. TS was determined from the list of *P* values obtained from the permutation tests for ME and CO associations in pathway, hallmark, and control pairs. We assumed these *P* values to be independently distributed such that the variance of TS can simply be estimated by 1 divided by the number of *P* values. The difference between two TSs follows a normal distribution, the mean of which can be estimated by the difference between the two TSs, and the standard deviation (SD) of which can be estimated by the sum of the two SD estimates. We took *P* values from the normal cumulative distribution function with this mean and SD to test for the difference in TSs between two groups. Groups were called significantly different when *P* ≤ 0.01.

### Software

Analyses were performed in MATLAB (MathWorks, Natick, MA, USA) and Python (open source).

## Results and discussion

### Mapping genes to pathways and to hallmarks

**Fig. 1:**
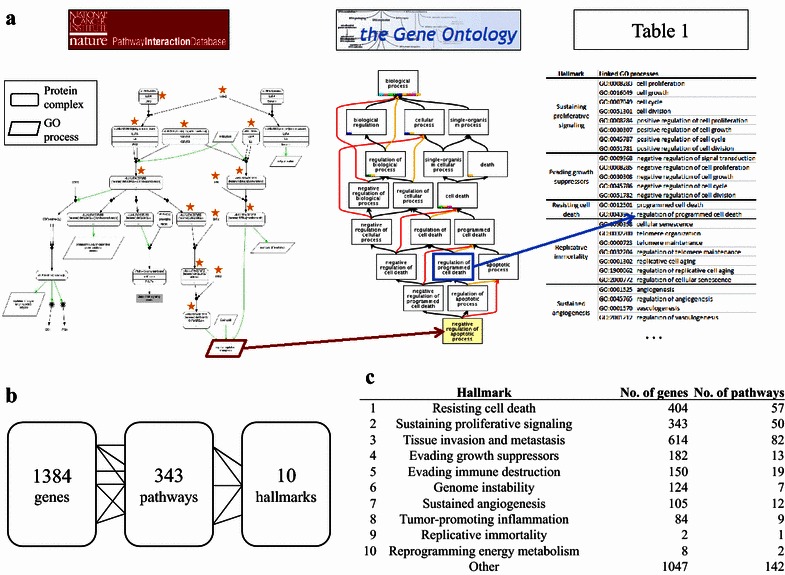
Multilevel map connecting genes via pathways to hallmarks. **a** Illustration of the automated process that links genes to pathways and to hallmarks. *Left* A pathway from the Pathway Interaction Database (PID). All genes marked with an orange star are upstream of the Gene ontology (GO) process “negative regulation of apoptotic process” (*red box*). (The text of the protein complexes and GO processes are not meant to be readable.) *Middle* A part of the GO hierarchy. The GO process “negative regulation of apoptotic process” is associated with the more general GO process “regulation of programmed cell death” (*blue box*), which is representative of the cancer hallmark “resisting cell death.” *Right* Mapping between each hallmark and one or more general GO terms. **b** Depiction of the multilevel map, which links 1384 genes to 343 pathways and to 10 hallmarks. **c** A table indicating the number of genes and pathways linked to each of the 10 hallmarks

We created a map that connects 1384 genes to 343 pathways and to the 10 cancer hallmarks (Fig. 1). On average, 200 genes signal to a hallmark via 25 pathways. However, there is a large variation in the number of genes and pathways connected to each of the hallmarks. For example, the hallmarks “resisting cell death,” “sustaining proliferative signaling,” and “tissue invasion and metastasis” are connected to, and thus possibly enabled by, 50 or more pathways and more than 300 genes. In contrast, the hallmarks “replicative immortality” and “reprogramming energy metabolism” are connected to 1 and 2 pathways, respectively, and only a handful of genes. Additionally, many GO processes in the PID could not be linked to any of the 10 cancer hallmarks. This resulted in 385 genes and 119 pathways that were solely connected to the category “Other” and were not considered further in this work.

Some well-known cancer genes are hubs in the map, i.e., they appear in many pathways and influence multiple hallmarks. For example, phosphatidylinositol-4,5-biphosphate 3-kinase, catalytic subunit alpha (*PIK3CA*) appears in 39 pathways and signals to 8 hallmarks, and tumor protein p53 (*TP53*) is found in 9 pathways and links to 4 hallmarks. However, the majority of genes are part of 1 or 2 pathways and link to 1 or 2 hallmarks. See Additional file 2: Figure S1, Additional file 3: Figure S2, Additional file 4: Figure S3, Additional file 5: Figure S4, Additional file 6: Figure S5 for a detailed graphical overview of the connectivity in this map.

### The multilevel mutational landscape

**Fig. 2:**
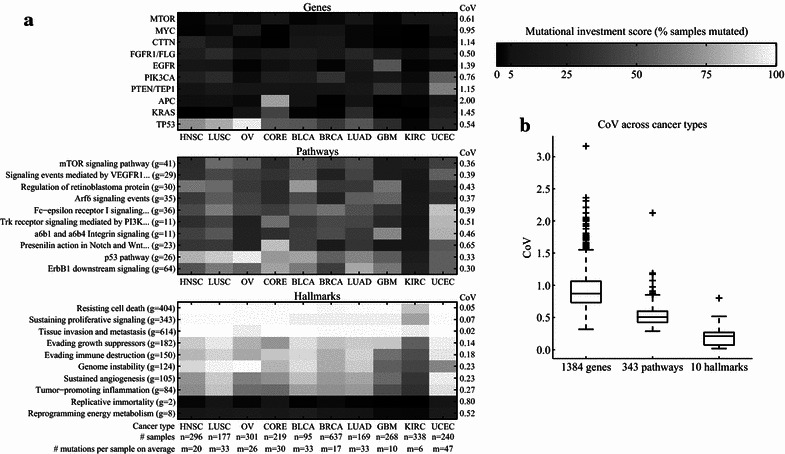
Mutation frequencies across cancer types. **a** Heatmaps depicting the percentage of samples within a cancer type that have a mutation in a gene (*top*), a mutation in at least one gene within a pathway (*middle*), and a mutation in at least one gene that is linked to a hallmark (*bottom*). Ten genes, 10 pathways, and all 10 hallmarks are shown. These genes and pathways were chosen based on the large variation in their mutation frequencies across cancer types. The coefficient of variation (CoV) in mutation frequencies across cancer types for each depicted gene, pathway, and hallmark is shown to the right of the heatmaps. Some pathway names are shortened for clarity. The number of genes in a pathway is stated in *parentheses* behind the pathway name. The number of genes linked to a hallmark is stated in *parentheses* behind the hallmark name. *MTOR* mechanistic target of rapamycin, *MYC* v-myc avian myelocytomatosis viral oncogene homolog, *CTTN* cortactin, *FGFR1/FLG* fibroblast growth factor receptor 1/filaggrin, *EGFR* epidermal growth factor receptor, *PIK3CA* phosphatidylinositol-4,5-bisphosphate 3-kinase, catalytic subunit alpha, *PTEN/TEP1* phosphatase and tensin homolog/telomerase-associated protein 1, *APC* adenomatous polyposis coli, *KRAS* Kirsten rat sarcoma viral oncogene homolog, *TP53* tumor protein p53, *HNSC* head and neck squamous cell carcinoma, *LUSC* lung squamous cell carcinoma, *OV* ovarian serous cystadenocarcinoma, *CORE* colon adenocarcinoma/rectum adenocarcinoma, *BLCA* bladder urothelial carcinoma, *BRCA* breast invasive carcinoma, *LUAD* lung adenocarcinoma, *GBM* glioblastoma multiforme, *KIRC* kidney renal clear cell carcinoma, *UCEC* uterine corpus endometrioid carcinoma. **b** *Boxplot* with the CoVs for all genes, pathways, and hallmarks

We projected the gene mutation data of the 2740 TCGA tumor samples onto the map. Grouping of the samples by their cancer types allowed us to investigate the average MI per cancer type at the level of genes, pathways, and hallmarks (Fig. 2a). At the level of genes, there was a large variation in mutation frequency among cancer types. This is not surprising because it is well known that cancer types are characterized by specific gene mutation signatures [12]. For example, adenomatous polyposis coli (*APC*) mutations are mostly specific to colorectal adenocarcinomas (CORE); 77% of these samples have an *APC* mutation, much more than the frequency for any other cancer type. At the level of pathways, there was a smaller variation in MI among cancer types. For example, the coefficient of variation (CoV) of the average MI score of the p53 pathway across cancer types was smaller than the CoV of the mutation status of the gene *TP53*. The CoV is defined as the SD divided by the mean. In this case, it is the SD of the 10 average MI scores divided by the mean of these 10 MI scores. At the level of hallmarks, the average MI scores across cancer types were even more similar to each other. Many of them were between 75 and 100%, indicating that for almost all tumor samples across cancer types there are gene mutations that link to each of the hallmarks. The two aforementioned sparsely connected hallmarks are exceptions. The greater similarity across cancer types at the level of hallmarks is also shown by the decrease of the average CoV from 0.93 to 0.53 to 0.25 when moving from genes to pathways to hallmarks (Fig. 2b).

We compared the CoVs derived from this map with those derived from 1000 randomly rewired multilevel maps (see “Randomly rewired map” in the Additional file 1 section). For these randomly rewired maps, genes were connected to randomly selected pathways, and pathways were connected to randomly selected hallmarks. However, the in-degree and out-degree of the two bipartite graphs in this map, i.e., from genes to pathways and from pathways to hallmarks, were maintained. We observed substantially higher CoVs for several pathways for the actual map compared with the randomized map (Additional file 7: Figure S6). This indicates that cancer types are characterized by different MI scores at the levels of not only genes but also pathways. Although the CoVs were small at the level of hallmarks, we found that for some hallmarks they were slightly yet consistently larger than those for randomly rewired maps, hinting that cancer types might have different MIs in hallmarks.

In conclusion, the multilevel map populated with mutation data establishes the already intuitive answer to our question of how genetically different tumors can share the same hallmark characteristics; mutations in different genes impinge on the same or functionally related pathways and ultimately deregulate the same biological processes.

### Mutual exclusivity and co-occurrence of gene mutations that enable hallmarks

Our strategy of propagating the binary mutation calls to the levels of pathways and hallmarks is based on the concept of “mutual exclusivity.” We investigated ME and CO genes in the context of the multilevel map.

**Fig. 3:**
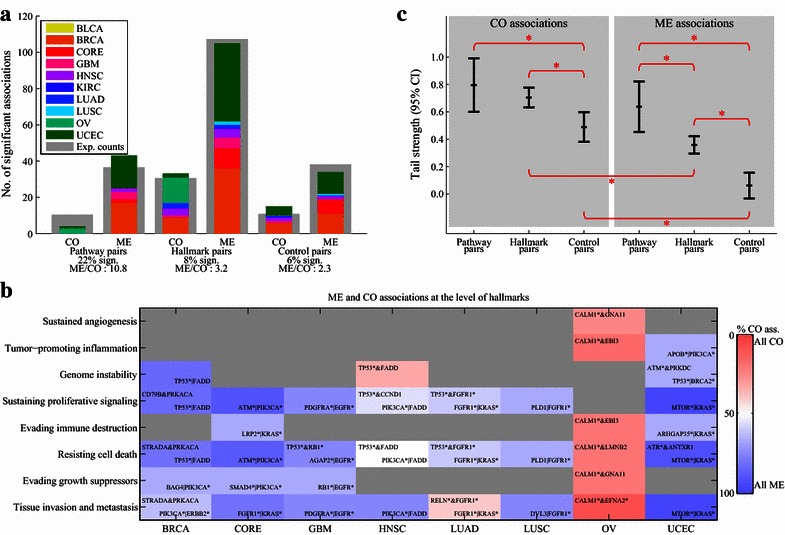
Mutually exclusive (ME) and co-occurring (CO) associations at the level of hallmarks. **a** *Bar diagram* depicting the total number of significant ME and CO associations found across all cancer types divided into three categories: pathway pairs, hallmark pairs, and control pairs. The *grey bars* in the background indicate the expected number of associations under the assumption that ME and CO associations are evenly distributed across the three categories. **b** Overview of ME and CO associations at the level of cancer hallmarks, i.e., associations found between pairs of genes that are not part of the same pathway but link to the same hallmark. The *color* indicates whether the majority of detected associations were ME (*blue*) or CO (*red*). The most significant ME association for each combination of a cancer type and a hallmark is printed in the *bottom-right corner of the box*, if available. Similarly, the most significant CO association is printed in the *top-left corner of the box*, if available. Genes with an *asterisk* are significantly frequently mutated in the respective cancer type as determined by the Mutational Significance in Cancer (MuSiC) or Genomic Identification of Significant Targets In Cancer (GISTIC), i.e., they are either frequently mutated or show recurrent copy number loss or gain. **c** Tail strengths (TSs) including the 95% confidence intervals (CIs) for the three categories: pathway pairs, hallmark pairs, and control pairs. Significant differences between TSs are indicated by a *red asterisk*. *CALM1* calmodulin 1 (phosphorylase kinase, delta), *GNA11* guanine nucleotide binding protein (G protein), alpha 11 (Gq class), *EBI3* Epstein-Barr virus induced 3, *APOB* apolipoprotein B, *FADD* Fas (TNFRSF6)-associated via death domain, *ATM* ATM serine/threonine kinase, *PRKDC* protein kinase, DNA-activated, catalytic polypeptide, *BRCA2* breast cancer 2, *CD79B* CD79b molecule, immunoglobulin-associated beta, *PRKACA* protein kinase, cAMP-dependent, catalytic, alpha, *PDGFRA* platelet-derived growth factor receptor, alpha polypeptide, *CCND1* cyclin D1, *FGFR1* fibroblast growth factor receptor 1, *PLD1* phospholipase D1, phosphatidylcholine-specific, *LRP2* low-density lipoprotein receptor-related protein 2, *ARHGAP35* Rho GTPase activating protein 35, *STRADA* STE20-related kinase adaptor alpha, *RB1* retinoblastoma 1, *AGAP2* ArfGAP with GTPase domain, ankyrin repeat and PH domain 2, *LMNB2* lamin B2, *ATR* ATR serine/threonine kinase, *ANTXR1* anthrax toxin receptor 1, *BAG4* BCL2-associated athanogene 4, *SMAD4* SMAD family member 4; ERBB2, erb-b2 receptor tyrosine kinase 2, *RELN* reelin, *DVL3* dishevel segment polarity protein 3, *EFNA2* ephrin-A2. Other abbreviations as in Fig. 2

The results of the statistical analyses showed that the largest number of significant ME and CO associations was discovered for pathway pairs (22% of all pathway pairs tested) followed by hallmark pairs (8%) and then control pairs (6%) (Fig. 3a). For all categories, we observed more ME associations than CO associations. However, the ratio of significant ME to CO associations differed substantially among the three categories (*P* = 0.03, Chi square test). Importantly, at the level of pathways, we observed more ME associations than CO associations (the ME:CO ratio was 11:1 for pathway pairs compared with an ME:CO ratio of 2:1 for control pairs), confirming the notion that mutual exclusivity is more prevalent in pathways. At the hallmark level, the ME:CO ratio was 3:1, which means that relative to the ME:CO ratio of 11:1 for pathways, there were more CO associations for the hallmark pairs. This observation hints that enabling hallmarks might require the concerted deregulation of distinct biological functions, yet not providing overwhelming evidence for this hypothesis.

This pattern was similar across most cancer types except for ovarian serous cystadenocarcinoma (OV), which was dominated by CO associations at the hallmark level (Fig. 3b). An overview of all significant associations is shown in Additional file 8: Table S1.

To investigate CO and ME associations further in pathways and hallmarks, we employed a complementary statistical analysis. Instead of looking only at the significant associations, we analyzed the complete distribution of *P* values using the TS statistic [20]. The TS and its confidence interval (CI) were obtained for the *P* values derived from the ME and CO tests for pathway, hallmark, and control pairs, separately (Fig. 3c). A high TS indicates that there are more small *P* values than is expected by chance. Interestingly, for hallmark and control pairs, the TS was significantly larger for CO associations than for ME associations. Thus, although there were more significant individual ME associations (Fig. 3a), the overall distribution of *P* values was skewed towards low *P* values for CO associations (Fig. 3c). Other observations of the TS were in line with the previous analysis. Specifically, the TS for ME associations was the highest in pathway pairs, followed by hallmark pairs, for which the TS was significantly lower. Control pairs had the lowest TS. For CO associations, in contrast, there was no significant difference in the TS between pathway and hallmark pairs. This provides further evidence for the hypothesis that ME is more prevalent in pathway pairs than in hallmark pairs.

### The role of infrequently mutated genes in pathway deregulation and hallmark activation

We employed the multilevel map to elucidate the role of genes that are not SFM in a cancer type.

First, we observed that many tumor samples (approximately 20% on average across all cancer types and hallmarks), which lacked mutations in SFM genes that were linked to hallmarks, had mutations in other genes that were linked to hallmarks and could potentially play a role in enabling them (Additional file 9: Figure S7).

**Fig. 4:**
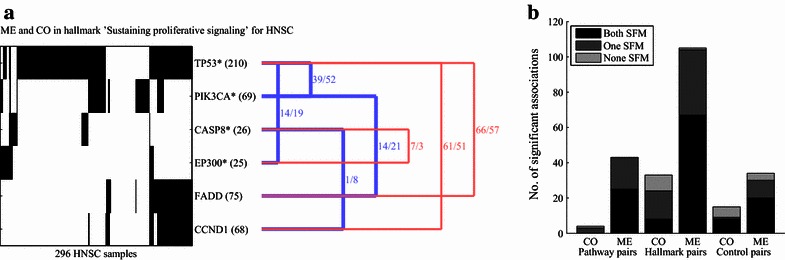
Infrequently mutated genes are part of ME and CO associations. **a** Mutations (depicted in *black*) in 6 genes that exhibited significant ME and CO associations in 296 HNSC samples. Genes with an *asterisk* are significantly frequently mutated (SFM). Numbers in *parentheses* behind the genes indicate the number of mutations. The *blue* and *red lines* to the right link ME and CO pairs, respectively. Numbers to the *right of lines* indicate the observed overlap (number of samples that have a mutation in both genes) followed by the randomly expected overlap. **b** *Bar diagram* similar to Fig. 3a, where CO and ME associations are divided into three groups: (1) both SFM, where both members of the pair are SFM; (2) one SFM, where one member is SFM; and (3) none SFM, where neither is SFM. CASP8, caspase 8; EP300, E1A-binding protein p300. Other abbreviations as in Figs. 2 and 3

Recently, ME and CO analysis was used to illuminate the role of these genes through associations with other genes in sub-networks [10]. Specifically, Leiserson et al. [10] identified sub-networks of ME and CO associations between genes using a protein–protein interaction network as a scaffold. In the present study, we used the multilevel map as a scaffold and identified many associations among genes that are not SFM. For example, the ME and CO analysis for head and neck squamous cell carcinoma (HNSC) revealed several associations between SFM and non-SFM genes (Fig. 4a). In general, almost half (45%) of all significant pairs at the pathway and hallmark levels included at least one gene that was not SFM in the tumor type (Fig. 4b). The distribution of pairs containing or consisting only of SFM genes was significantly skewed across ME and CO associations (*P* < 0.001, Chi square test) as well as across the three categories (*P* < 0.001, Chi square test). In addition, we found a large number of CO pairs at the hallmark level containing one or no SFM genes. This is a clear indication that the concerted effect of mutations in genes that are not SFM could play an important role in establishing cancer hallmark characteristics.

## Conclusions

We have created a map that connects genes to cancer hallmarks via pathways. We projected gene mutation and focal copy number alteration data from various cancer types onto this map. This allowed us to show that ME gene mutations are more prevalent in pathways compared with hallmarks, and conversely, that CO gene mutations are relatively important to enable hallmark characteristics. In addition, we demonstrated how the multilevel map can help to clarify the role of infrequently mutated genes.

However, making relevant predictions of how molecular events affect cellular and tissue phenotypes will involve computational multilevel models that are much richer in biological knowledge and data than the model presented here. We have employed a straightforward strategy to project the cancer gene mutation and copy number data onto the multilevel map. Future approaches will have to balance the added benefit of integrating additional knowledge and data with the added model complexity. We foresee several approaches that would yield more functional insight from the multilevel map when integrated with molecular data. First, information about genes can be formalized within the map. For example, many cancer genes can be classified as either tumor suppressor genes, which are inactivated by mutations or deletions, or oncogenes, which are activated by mutations or amplifications. These distinct roles will help to predict the downstream consequences of genomic aberrations. In addition, the position of a mutation in a gene potentially has an effect on its functional consequences [21], which could be taken into account. Second, currently unused information in the PID about the interactions between proteins (and other biomolecules), including positive and negative regulation, protein–protein binding, and other interactions, would enable the inference of regulatory relationships instead of simple statistical associations. Third, integration of additional molecular data, including gene and protein expression as well as epigenetic modifications, would make the information flow across the map more physiologically relevant. There are already some approaches that integrate different data types and interactions in a systematic and quantitative way [22, 23]. However, none of these approaches explicitly incorporate hallmarks into their framework.

In its current form, there are several important considerations about the multilevel map. First, the map is incomplete in terms of genes. A few frequently mutated genes, such as AT rich interactive domain 1A (*ARID1A*) and mucin 16 (*MUC16*), are not part of the map, simply because they are not part of PID. The use of pathway databases not only prevents the discovery of novel, relevant genes, but also limits the use of more recently discovered, and thus less studied, cancer genes. Second, certain cancer hallmarks are poorly characterized as evidenced by the very small number of genes and pathways that could be linked to these hallmarks. This seeming lack of annotation might be salvaged by updated pathway information. In addition, the large number of GO categories in the PID that could not be associated with cancer hallmarks could be revisited specifically with these underrepresented hallmarks in mind. Third, the map lacks certain well-established relationships. Specifically, the automated procedure was not able to map some well-known cancer genes, including cyclin-dependent kinase inhibitor 2A (*CDKN2A*), F-box and WD repeat domain containing 7 (*FBXW7*), and E2F transcription factor 3 (*E2F3*), to a hallmark. Although common knowledge would link the cell cycle regulator *CDKN2A* to sustained proliferative signaling and perhaps other hallmarks, these relations were not present as such in the PID and the GO.

Despite these shortcomings, we conjecture that multilevel maps, such as the one presented here, will help in the interpretation of large cancer genomic data sets. The use of three functional levels, i.e., genes, pathways, and hallmarks, facilitates an intuitive understanding. The pathways and hallmarks can be seen as conceptual tools that represent the functional levels crucial for an intelligible mapping from genes to the phenotype. The information in the map is easily assimilated with the researcher’s domain knowledge empowering the formulation of novel hypotheses and experiments.

## Additional files

Additional file 1. Supplementary methods and results. This document contains supplementary text about the multilevel map, the mutation data, the significantly mutated and altered copy number genes, and the randomly rewired map. Additional file 2:
**Figure S1.** Connectivity in the map between genes and pathways. The heatmap indicates the membership (black squares) of genes within pathways. The grey bars to the right indicate the number of genes that are part of a pathway. The grey bars on the top indicate the number of pathways that a gene is a member of. Additional file 3:
**Figure S2.** Connectivity in the map between pathways and hallmarks. The heatmap indicates the membership (black squares) of pathways within hallmarks. The grey bars to the right indicate the number of pathways that are linked to a hallmark. The grey bars on the top indicate the number of hallmarks that a pathway is pointing to. Additional file 4:
**Figure S3.** Connectivity in the map between genes and hallmarks. The heatmap indicates the membership (black squares) of genes within hallmarks. The grey bars to the right indicate the number of genes that are linked to a hallmark. The grey bars on the top indicate the number of hallmarks that a gene is pointing to. Additional file 5:
**Figure S4.** Connectivity in the map between the 25 most frequently mutated genes and pathways. Similar to Additional file 2: Figure S1 but showing the connectivity for only the 25 most frequently mutated genes. E2F3, E2F transcription factor 3; RB1, retinoblastoma 1; CCND1, cyclin D1; CDKN2A, cyclin-dependent kinase inhibitor 2A; APOB, apolipoprotein B; TNIK, TRAF2 and NCK interacting kinase; LRP2, low-density lipoprotein receptor-related protein 2; EPHB3, EPH receptor B3; FBXW7, F-box and WD repeat domain containing 7; DVL3, dishevel segment polarity protein 3; RELN, reelin; APC, adenomatous polyposis coli; NOTCH1, notch 1; FGFR1/FLG, fibroblast growth factor receptor 1/filaggrin; FADD, Fas (TNFRSF6)-associated via death domain; PTEN/TEP1, phosphatase and tensin homolog/telomerase-associated protein 1; CTTN, cortactin; EGFR, epidermal growth factor receptor; PLD1, phospholipase D1, phosphatidylcholine-specific; TP53, tumor protein p53; EP300, E1A-binding protein p300; KRAS, Kirsten rat sarcoma viral oncogene homolog; CTNNB1, catenin (cadherin-associated protein), beta 1; PIK3CA, phosphatidylinositol-4,5-bisphosphate 3-kinase, catalytic subunit alpha; PIK3R1, phosphoinositide-3-kinase, regulatory subunit 1 (alpha). Additional file 6:
**Figure S5.** Connectivity in the map between the 25 most frequently mutated genes and hallmarks. Similar to Additional file 4: Figure S3 but showing the connectivity for only the 25 most frequently mutated genes. Abbreviations as in Additional file 5: Figure S4. Additional file 7:
**Figure S6**. The coefficient of variation (CoV) across cancer types at the gene, pathway, and hallmark levels. A, violin plots of the CoV of the average mutational investment (MI) scores for the genes, pathways, and hallmarks across the ten cancer types. The black violin plots are based on the actual map, whereas the red plots are the average values across 1,000 randomly rewired maps. B, scatterplots of CoV (top panels), standard deviation (SD) (middle panels), and means (bottom panels) for pathways (left panels) and hallmarks (right panels) comparing the actual values (x-axis, as also depicted in A) with the average values across 1,000 randomly rewired maps (y-axis). Data points from the actual map that are more than 2SD away from the mean of the 1,000 randomly rewired maps are highlighted with circles and squares. In addition, the total number of data points that are more than 2SD larger or smaller than random are printed above the figure. Additional file 8:
**Table S1.** Significant mutually exclusive (ME) and co-occurring (CO) associations across all cancer types. Genes with an asterisk (*) are significantly frequently mutated as determined by the Mutational Significance in Cancer (MuSiC) and/or Genomic Identification of Significant Targets In Cancer (GISTIC). Additional file 9:
**Figure S7**. Mutational investment in hallmarks explained by significantly mutated genes and non-significantly mutated genes. For each combination of a cancer type and a hallmark, blue areas indicate the percentage of samples that have a mutation in at least one gene that is linked to the hallmark and significantly frequently mutated according to Mutational Significance in Cancer (MuSiC) and/or Genomic Identification of Significant Targets In Cancer (GISTIC); grey areas indicate the percentage of samples that have a mutation in at least one gene that is linked to the hallmark, but none of these samples have a mutation in a significantly frequently mutated gene; black areas indicate the percentage of samples that have no mutated genes that link to the hallmark. BLCA, bladder urothelial carcinoma, BRCA, breast invasive carcinoma; CORE, colon/rectum adenocarcinoma; GBM, glioblastoma multiforme; HNSC, head and neck squamous cell carcinoma; KIRC, kidney renal clear cell carcinoma; LUAD, lung adenocarcinoma; LUSC, lung squamous cell carcinoma; OV, ovarian serous cystadenocarcinoma; UCEC, uterine corpus endometrioid carcinoma.
