# Supplementary material for: A multilevel pan-cancer map links gene mutations to cancer hallmarks
Source: Chin J Cancer. 2015 Sep 14;34:48. doi: 10.1186/s40880-015-0050-6 (PMC4593384; doi:10.1186/s40880-015-0050-6)
Supplement: Additional file 1. — Supplementary methods and results. This document contains supplementary text about the multilevel map, the mutation data, the significantly mutated and altered copy number genes, and the randomly rewired map. [file 40880_2015_50_MOESM1_ESM.docx]

**Additional File 1 - Supplementary Methods and Results.**

This document contains supplementary text about the multilevel map, the mutation data, the significantly mutated and altered copy number genes, and the randomly rewired map.

**Supplementary Methods**

All data can be found at Synapse.org with identifier syn4216888 (http://dx.doi.org/10.7303/syn4216888).

**Multilevel map**

We constructed a three-level mapping connecting 1,384 genes to 343 pathways and 10 hallmarks. The mapping from genes to pathways was constructed based on information from the Pathway Interaction Database (PID, http://pid.nci.nih.gov/) [1]. The mapping from pathways to hallmarks was constructed from the Gene Ontology (GO, http://geneontology.org/) [2]. This resulted in a multilevel graph that connects biological entities of different levels of functional abstraction, i.e., genes, pathways, and hallmarks.

For the mapping from genes to pathways, we searched the PID for proteins that are in a signaling pathway that signals toward a GO biological process. These associations were automatically extracted using Simple Protocol and RDF Query Language (SPARQL) [3] queries from a Sesame 2.7 triple store (ftp://ftp1.nci.nih.gov/pub/PID/BioPAX_Level_3/NCI-Nature_Curated.bp3.owl.gz), which contains the latest version of the PID in Biopax 3 format [4]. A query for proteins signaling to a GO biological process follows the signaling pathway from molecules directly regulating the GO processes through the biochemical reactions. All proteins encountered along the signaling pathway, including the ones within a complex or protein family, are associated with the signaling pathway. The biochemical reaction network of the PID is divided into 167 pathways. Each PID pathway was manually created by a group of experts. We queried every PID pathway separately. Specifically, only those proteins that are part of the PID pathway and can directly (via biochemical reactions) affect the GO process are part of the signaling pathway. We used the name of the PID pathway as the name of the group of proteins found in the signaling pathway. The 818 signaling pathways that were found in this way were reduced to 343 because many signaling pathways originate from the same PID pathway and are (almost) identical in terms of gene content. In most cases, such pairs of pathways only differ in the GO process towards which they signal. After the merging step, each of these 343 pathways signals to one or more GO processes.

For the mapping from pathways to hallmarks, we employed the hierarchy in GO, specifically using the “is a” and “part of” relationships. We manually associated each of the hallmarks of cancer with one or more general GO terms in this hierarchy (**Table 1**). This process was performed by the authors with the help of domain experts at the Netherlands Cancer Institute. For the GO processes, which are associated with the pathways, we automatically checked whether they fall under one of the general GO terms, by using the “is a” and “part of” relationships. Using this strategy, pathways were associated with the cancer hallmarks. GO processes that could not be linked to any of the 10 cancer hallmarks were linked to hallmark “Other.”

All data and scripts pertaining to the multilevel map can be downloaded at Synapse.org with the identifier syn4216890 (http://dx.doi.org/10.7303/syn4216890).

**Mutation data**

The mutation data are represented as a binary matrix with 1,384 genes and 2,740 samples from 10 different tumor types. A gene is called mutated in a TCGA sample if it meets one (or both) of these conditions: 1) a non-silent mutation is listed in the Pancan12 mutation annotation file (MAF, pancan12_cleaned.maf), which can be found at Synapse.org with the identifier syn1710680 (http://dx.doi.org/10.7303/syn1710680.4); 2) the gene is focally amplified or deleted. Copy number variation data were obtained from Broad’s Firehose GISTIC run at http://gdac.broadinstitute.org/runs/analyses__2013_02_22/reports/cancer/PANCAN12/CopyNumber_Gistic2/nozzle.html (http://dx.doi.org/10.7908/C1JH3JDB). These data can also be found at Synapse.org with the identifier 1703357 (http://dx.doi.org/10.7303/syn1703357).

A gene is called focally amplified when its copy number is larger than 2 and focally deleted when its copy number is smaller than -1. We used the data in the file focal_data_by_genes.pancan12.txt on the Synapse page.

The binary gene, pathway, and hallmark (mutation investment) scores as well as annotations for the genes, pathways, and hallmarks and the connections in the multilevel map can be downloaded at Synapse.org with the identifier syn4216891 (http://dx.doi.org/10.7303/syn4216891).

**Significantly mutated and altered copy number genes**

A list of significantly mutated genes called by the Mutational Significance in Cancer (MuSiC) algorithm [5] for each cancer type was obtained from the _smgs.tsv files from https://www.synapse.org/#!Synapse:syn1713813.

A list of focally amplified and deleted genes called by the Genomic Identification of Significant Targets in Cancer (GISTIC) algorithm [6] for each cancer type was obtained from the _amp.txt and _del.txt files from https://www.synapse.org/#!Synapse:syn1713807.

**Random map rewiring**

To create a randomly rewired multilevel map, we applied BiRewire [7] on two binary matrices: 1) the genes to pathway membership matrix (dimensions: 1384 genes by 343 pathways) and 2) the pathway to hallmark membership matrix (dimensions: 343 pathways by 10 hallmarks).

**Supplementary Results**

**Randomly rewired map**

This rewiring retained the overall topological structure and mutation landscape. Specifically, after rewiring, each gene still had the same mutation rate as in the actual mutation matrix. Consequently, at the gene level the coefficient of variation (CoV), standard deviation (SD), and means were identical between the actual and randomized map (**Additional File 7: Figure S6**). The in-degree and out-degree of the genes, pathways, and hallmarks in the multilevel map remained the same after rewiring. For example, in the randomized map, the gene tumor protein p53 (*TP53*) was part of the same number of pathways (randomly selected) as in the actual map. In addition, the P53 pathway contained the same number of genes (randomly selected) as in the actual map. We created 1000 randomly rewired maps for the experiments described in **Additional File 7: Figure S6.**

**References**

1. Schaefer CF, Anthony K, Krupa S, Buchoff J, Day M, Hannay T, et al. PID: the Pathway Interaction Database. Nucleic Acids Res. 2009;37(Database issue):D674-9.

2. Ashburner M, Ball CA, Blake JA, Botstein D, Butler H, Cherry JM, et al. Gene ontology: tool for the unification of biology. Nat Genet. 2000;25(1):25-9.

3. Harris S, Seaborne A. SPARQL 1.1 query language. W3C Recommendation. 2013. Available at: http://www.w3.org/TR/sparql11-query/. Accessed on: May 2013.

4. Demir E, Cary MP, Paley S, Fukuda K, Lemer C, Vastrik I, et al. The BioPAX community standard for pathway data sharing. Nat Biotechnol. 2010;28(9):935-42.

5. Dees ND, Zhang Q, Kandoth C, Wendl MC, Schierding W, Koboldt DC, et al. MuSiC: identifying mutational significance in cancer genomes. Genome Res. 2012;22(8):1589-98.

6. Mermel CH, Schumacher SE, Hill B, Meyerson ML, Beroukhim R, Getz G. GISTIC2.0 facilitates sensitive and confident localization of the targets of focal somatic copy-number alteration in human cancers. Genome Biol. 2011;12(4):R41.

7. Gobbi A, Iorio F, Dawson KJ, Wedge DC, Tamborero D, Alexandrov LB, et al. Fast randomization of large genomic datasets while preserving alteration counts. Bioinformatics. 2014;30(17):i617-23.
