# Supplementary figures and images for: A multilevel pan-cancer map links gene mutations to cancer hallmarks
Source: Chin J Cancer. 2015 Sep 14;34:48. doi: 10.1186/s40880-015-0050-6 (PMC4593384; doi:10.1186/s40880-015-0050-6)

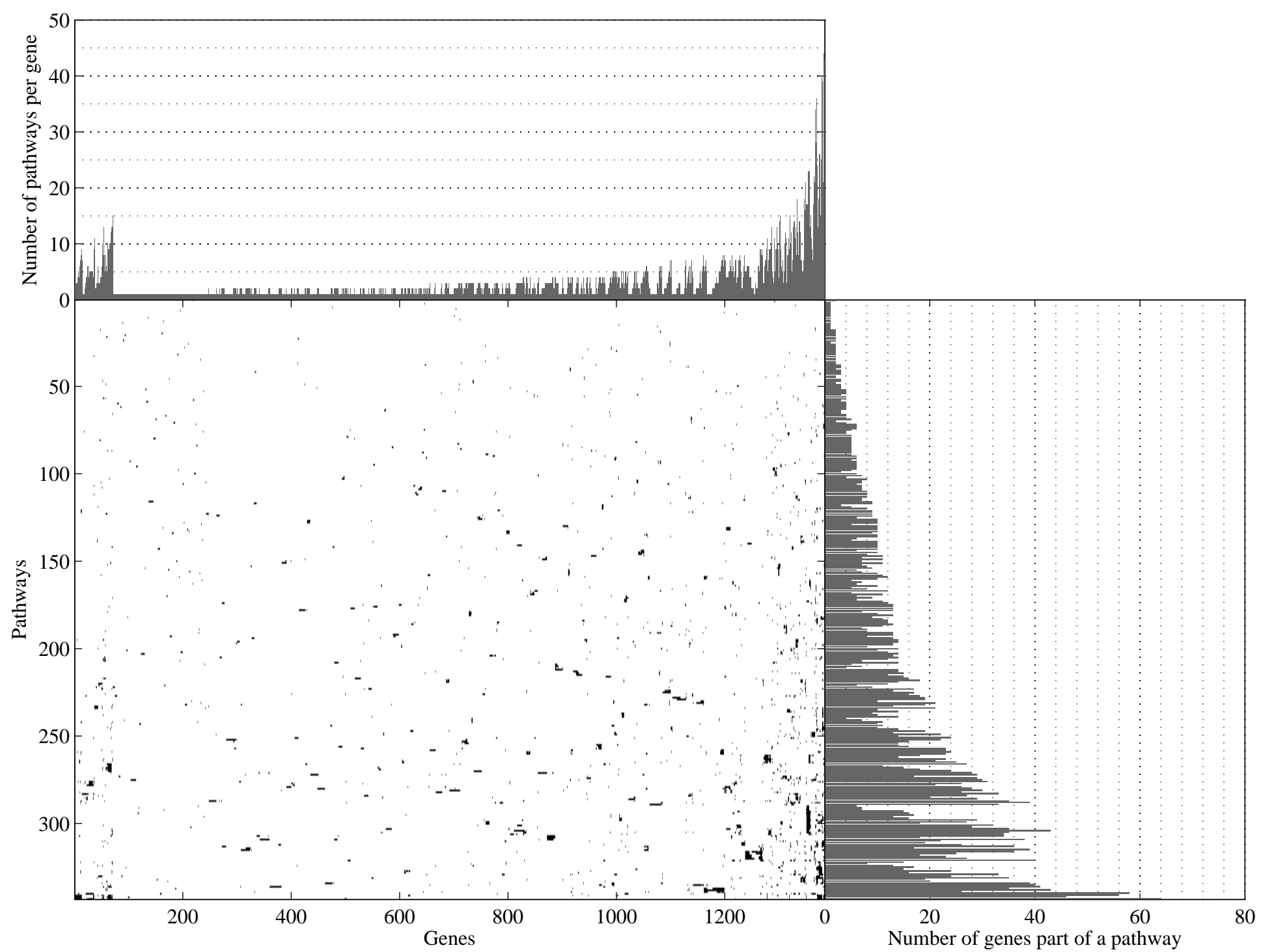

Supplement: Additional file 2: — Figure S1. Connectivity in the map between genes and pathways. The heatmap indicates the membership (black squares) of genes within pathways. The grey bars to the right indicate the number of genes that are part of a pathway. The grey bars on the top indicate the number of pathways that a gene is a member of. [file 40880_2015_50_MOESM2_ESM.pdf]

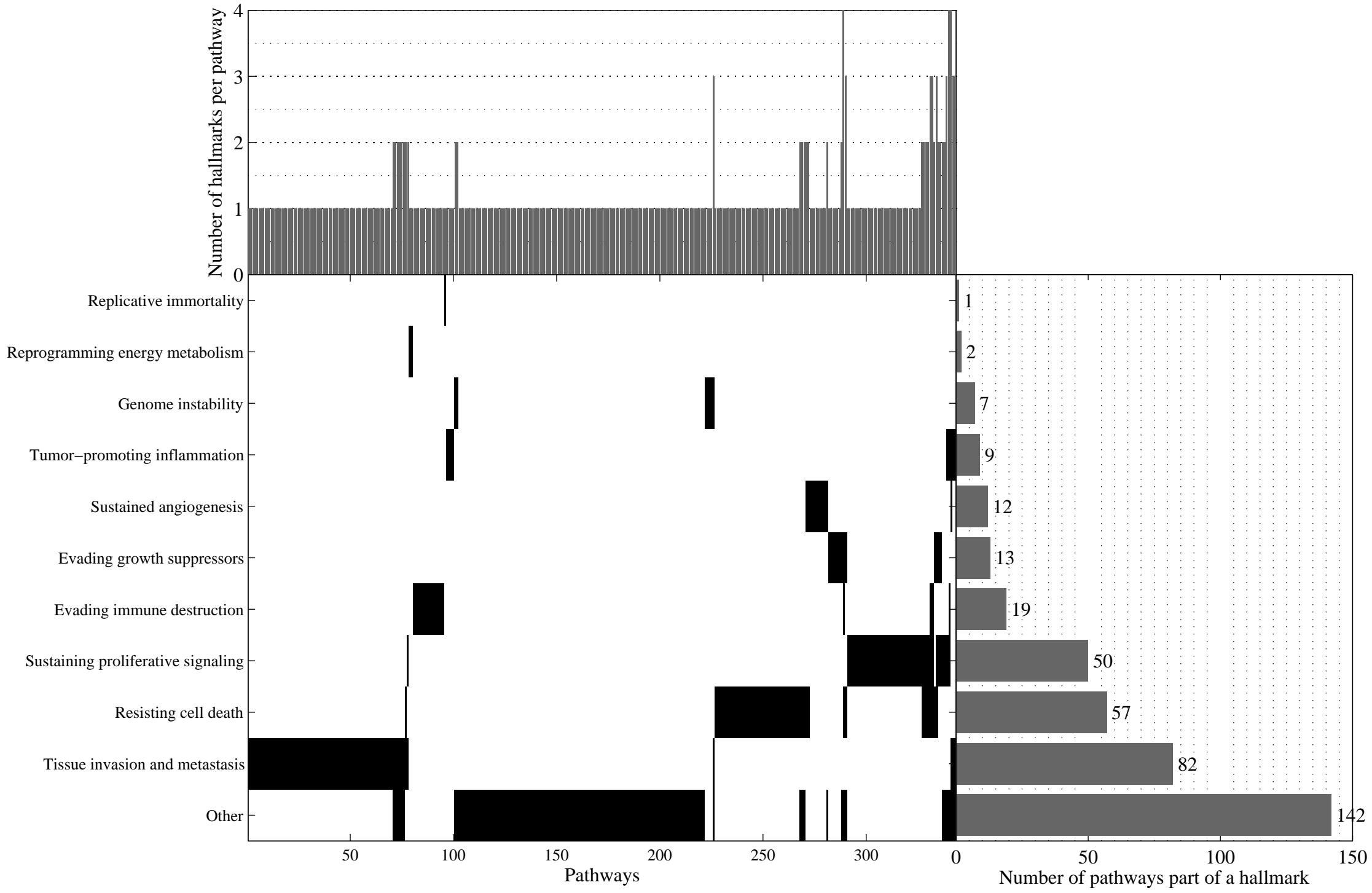

Supplement: Additional file 3: — Figure S2. Connectivity in the map between pathways and hallmarks. The heatmap indicates the membership (black squares) of pathways within hallmarks. The grey bars to the right indicate the number of pathways that are linked to a hallmark. The grey bars on the top indicate the number of hallmarks that a pathway is pointing to. [file 40880_2015_50_MOESM3_ESM.pdf]

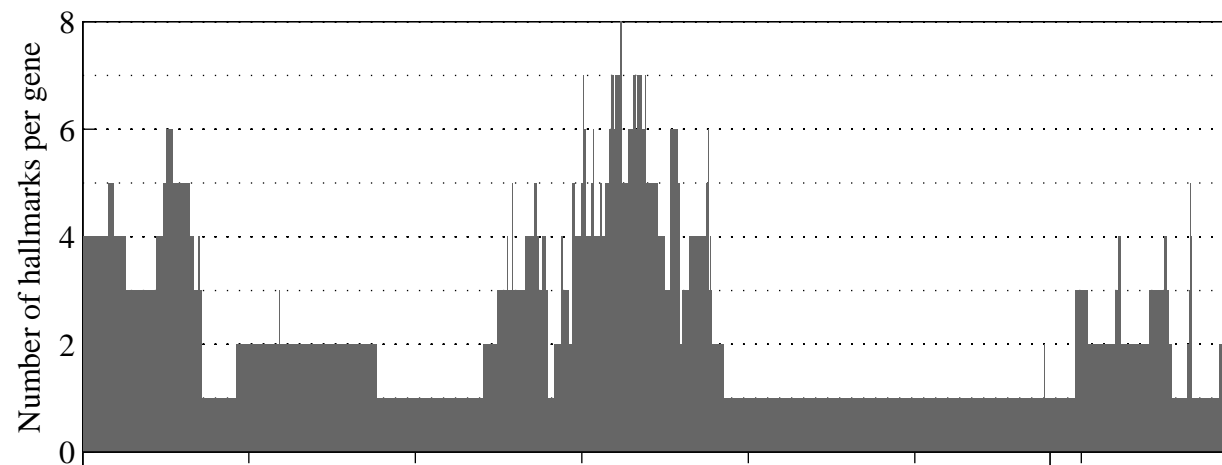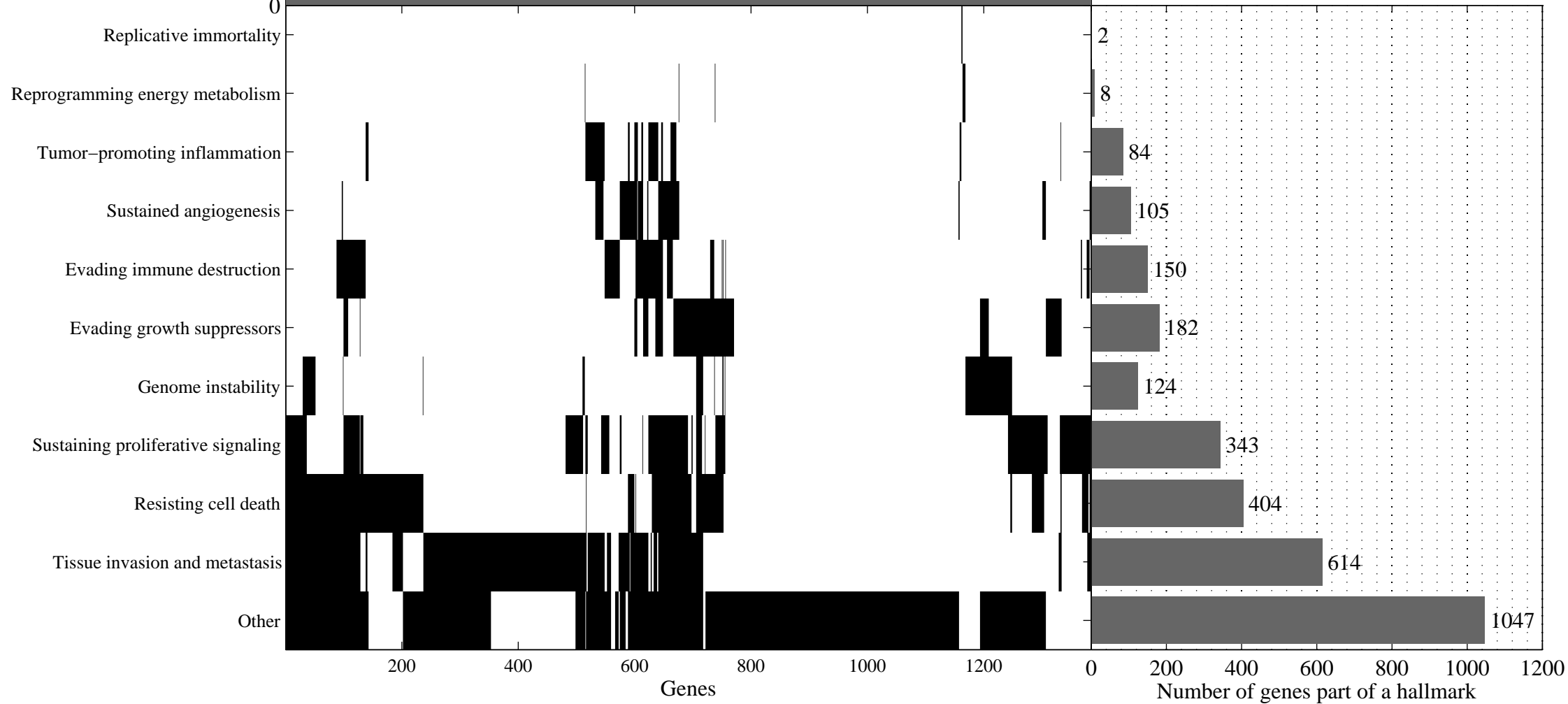

Supplement: Additional file 4: — Figure S3. Connectivity in the map between genes and hallmarks. The heatmap indicates the membership (black squares) of genes within hallmarks. The grey bars to the right indicate the number of genes that are linked to a hallmark. The grey bars on the top indicate the number of hallmarks that a gene is pointing to. [file 40880_2015_50_MOESM4_ESM.pdf]

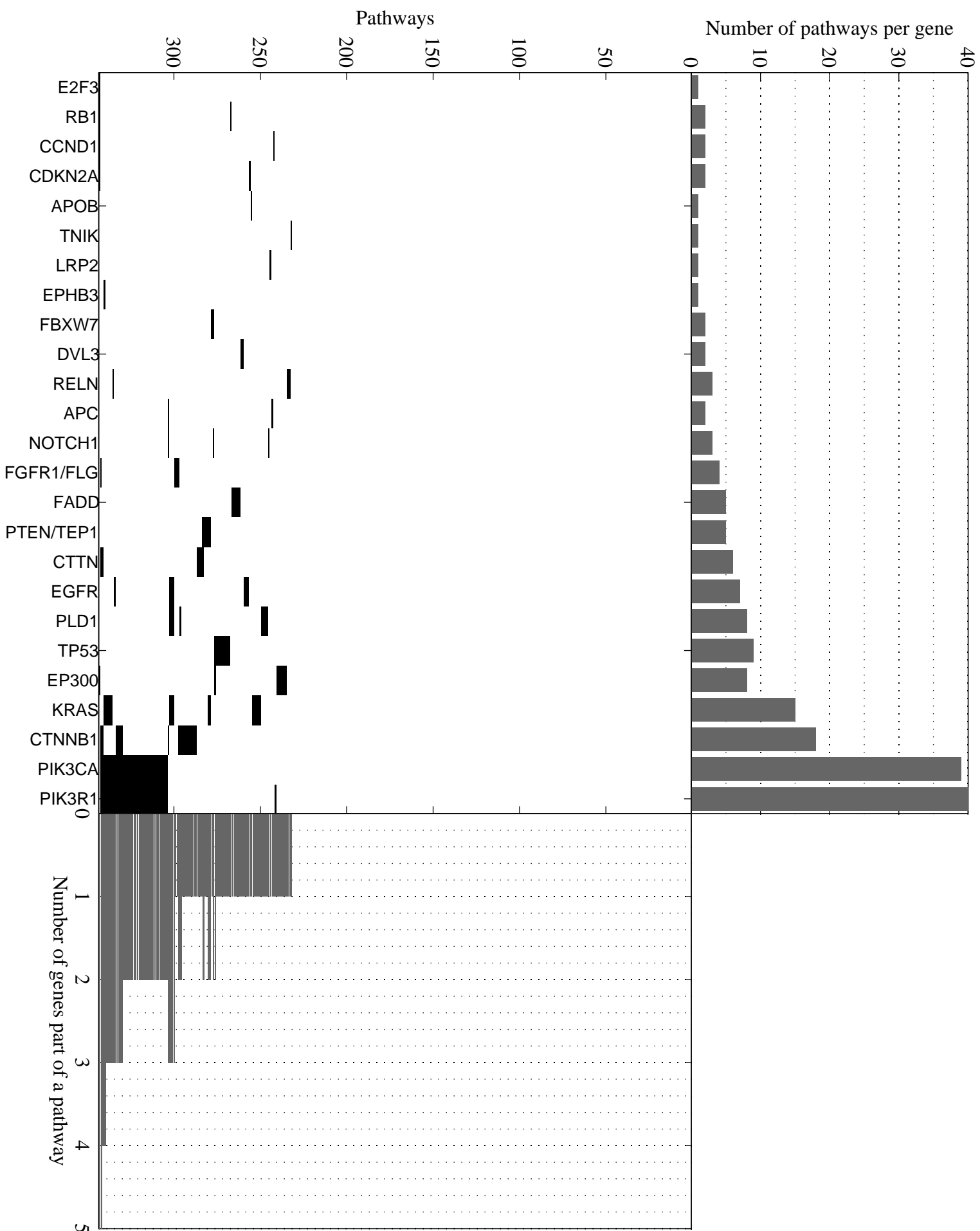

Supplement: Additional file 5: — Figure S4. Connectivity in the map between the 25 most frequently mutated genes and pathways. Similar to Additional file 2: Figure S1 but showing the connectivity for only the 25 most frequently mutated genes. E2F3, E2F transcription factor 3; RB1, retinoblastoma 1; CCND1, cyclin D1; CDKN2A, cyclin-dependent kinase inhibitor 2A; APOB, apolipoprotein B; TNIK, TRAF2 and NCK interacting kinase; LRP2, low-density lipoprotein receptor-related protein 2; EPHB3, EPH receptor B3; FBXW7, F-box and WD repeat domain containing 7; DVL3, dishevel segment polarity protein 3; RELN, reelin; APC, adenomatous polyposis coli; NOTCH1, notch 1; FGFR1/FLG, fibroblast growth factor receptor 1/filaggrin; FADD, Fas (TNFRSF6)-associated via death domain; PTEN/TEP1, phosphatase and tensin homolog/telomerase-associated protein 1; CTTN, cortactin; EGFR, epidermal growth factor receptor; PLD1, phospholipase D1, phosphatidylcholine-specific; TP53, tumor protein p53; EP300, E1A-binding protein p300; KRAS, Kirsten rat sarcoma viral oncogene homolog; CTNNB1, catenin (cadherin-associated protein), beta 1; PIK3CA, phosphatidylinositol-4,5-bisphosphate 3-kinase, catalytic subunit alpha; PIK3R1, phosphoinositide-3-kinase, regulatory subunit 1 (alpha). [file 40880_2015_50_MOESM5_ESM.pdf]

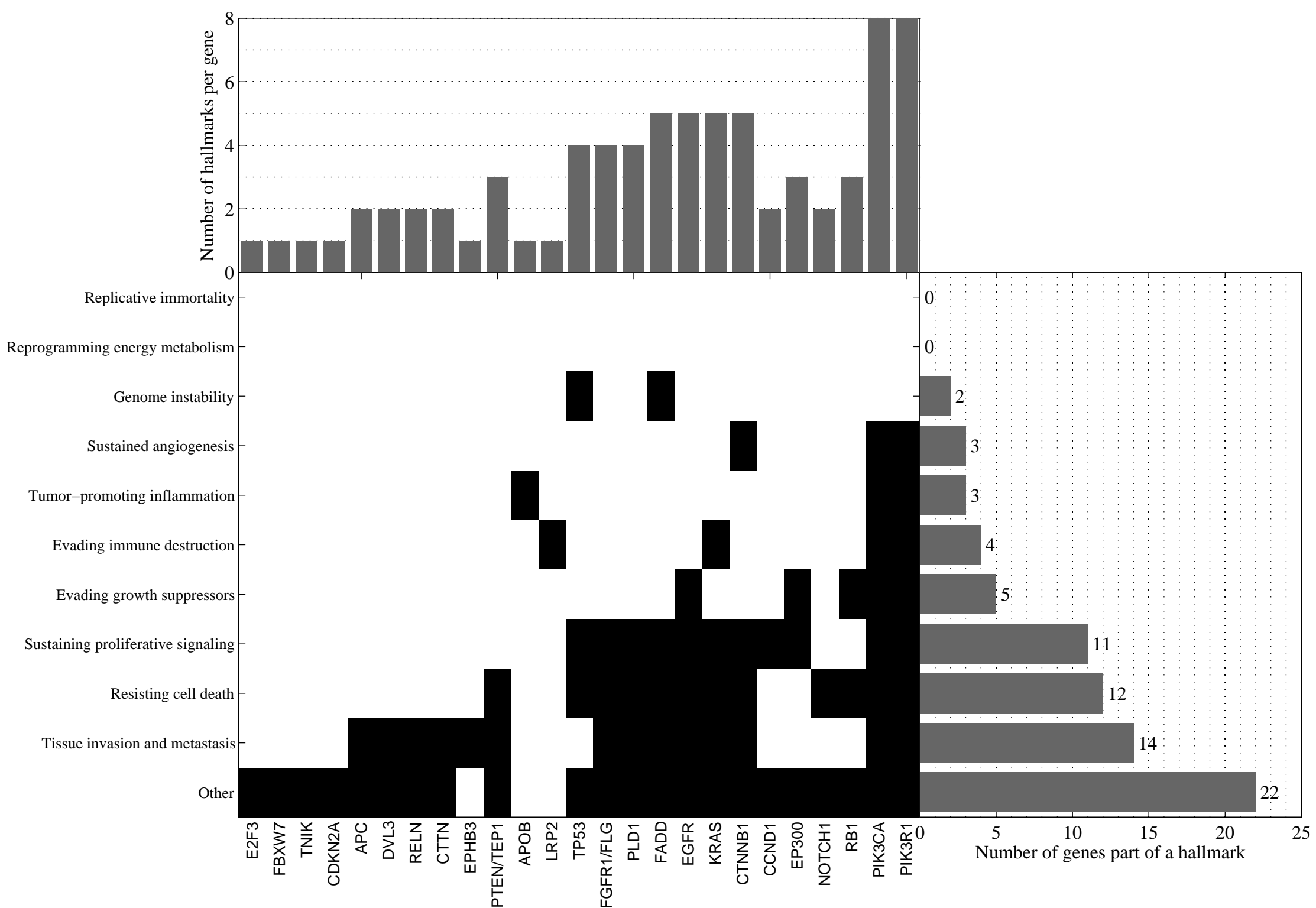

Supplement: Additional file 6: — Figure S5. Connectivity in the map between the 25 most frequently mutated genes and hallmarks. Similar to Additional file 4: Figure S3 but showing the connectivity for only the 25 most frequently mutated genes. Abbreviations as in Additional file 5: Figure S4. [file 40880_2015_50_MOESM6_ESM.pdf]

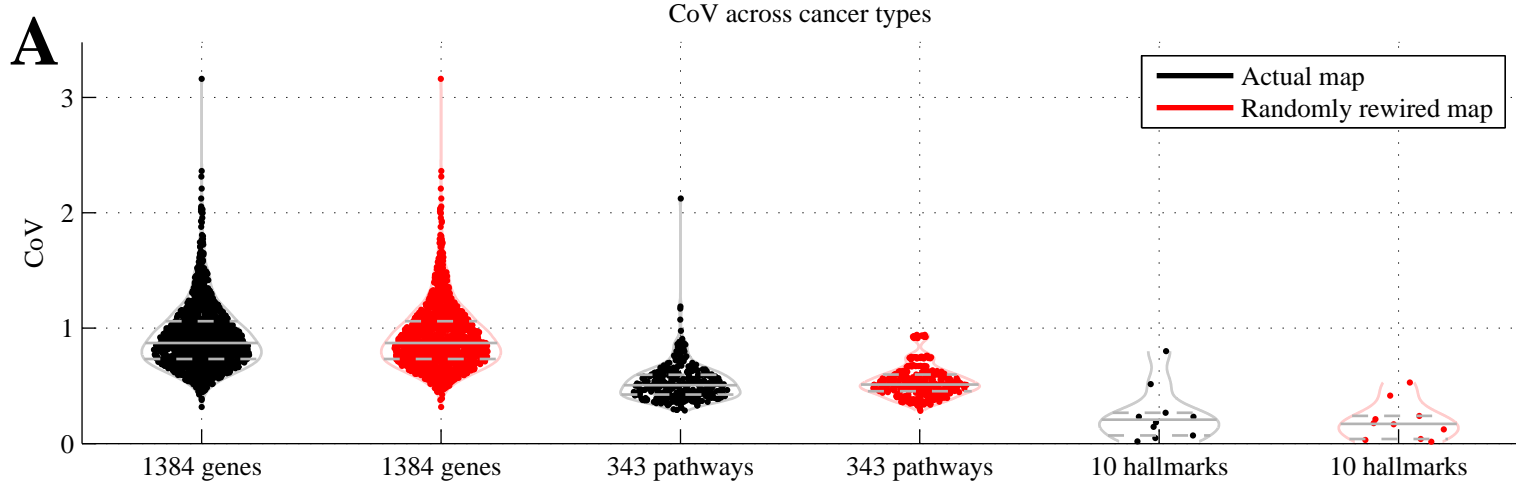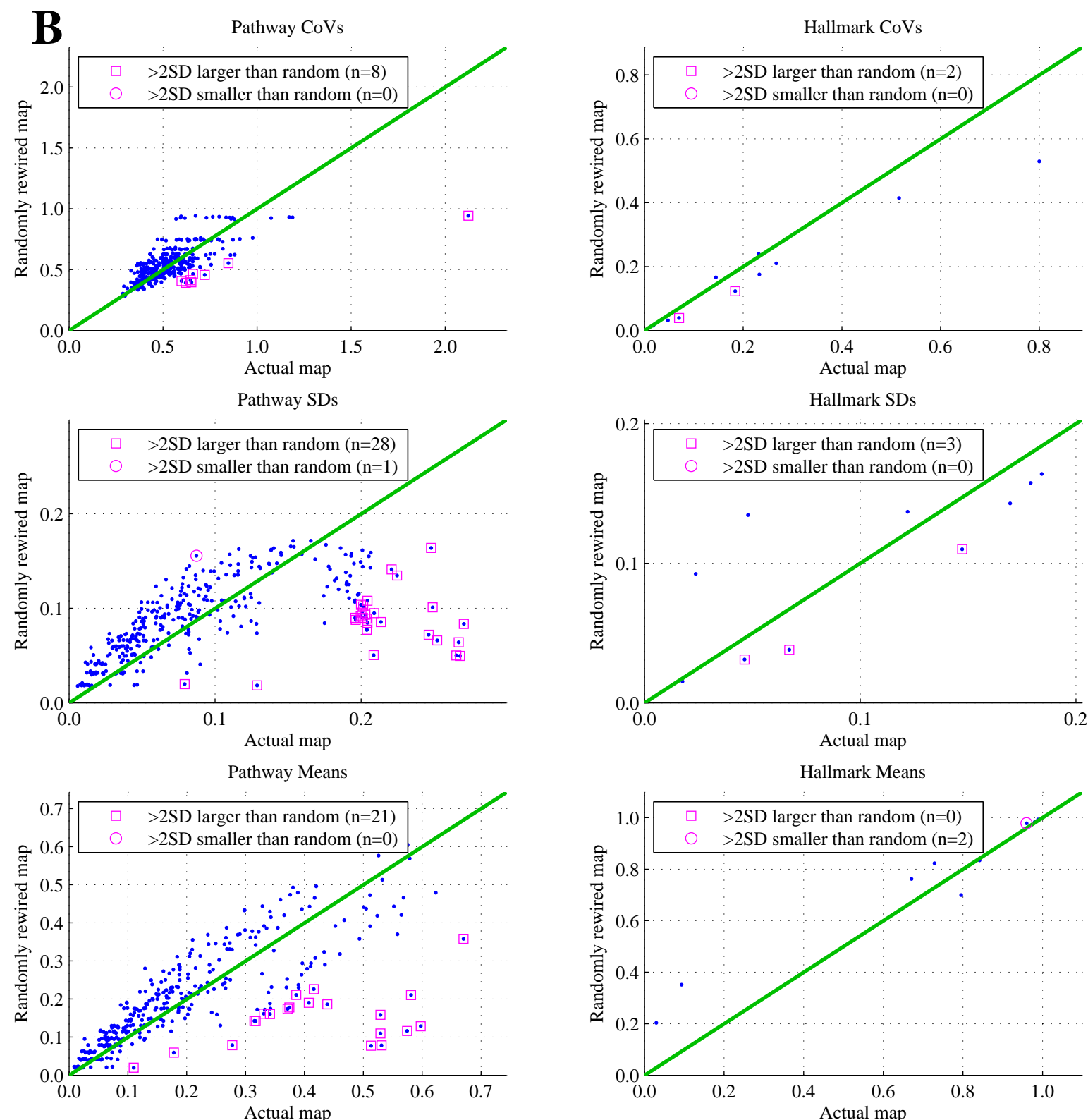

Supplement: Additional file 7: — Figure S6. The coefficient of variation (CoV) across cancer types at the gene, pathway, and hallmark levels. A, violin plots of the CoV of the average mutational investment (MI) scores for the genes, pathways, and hallmarks across the ten cancer types. The black violin plots are based on the actual map, whereas the red plots are the average values across 1,000 randomly rewired maps. B, scatterplots of CoV (top panels), standard deviation (SD) (middle panels), and means (bottom panels) for pathways (left panels) and hallmarks (right panels) comparing the actual values (x-axis, as also depicted in A) with the average values across 1,000 randomly rewired maps (y-axis). Data points from the actual map that are more than 2SD away from the mean of the 1,000 randomly rewired maps are highlighted with circles and squares. In addition, the total number of data points that are more than 2SD larger or smaller than random are printed above the figure. [file 40880_2015_50_MOESM7_ESM.pdf]

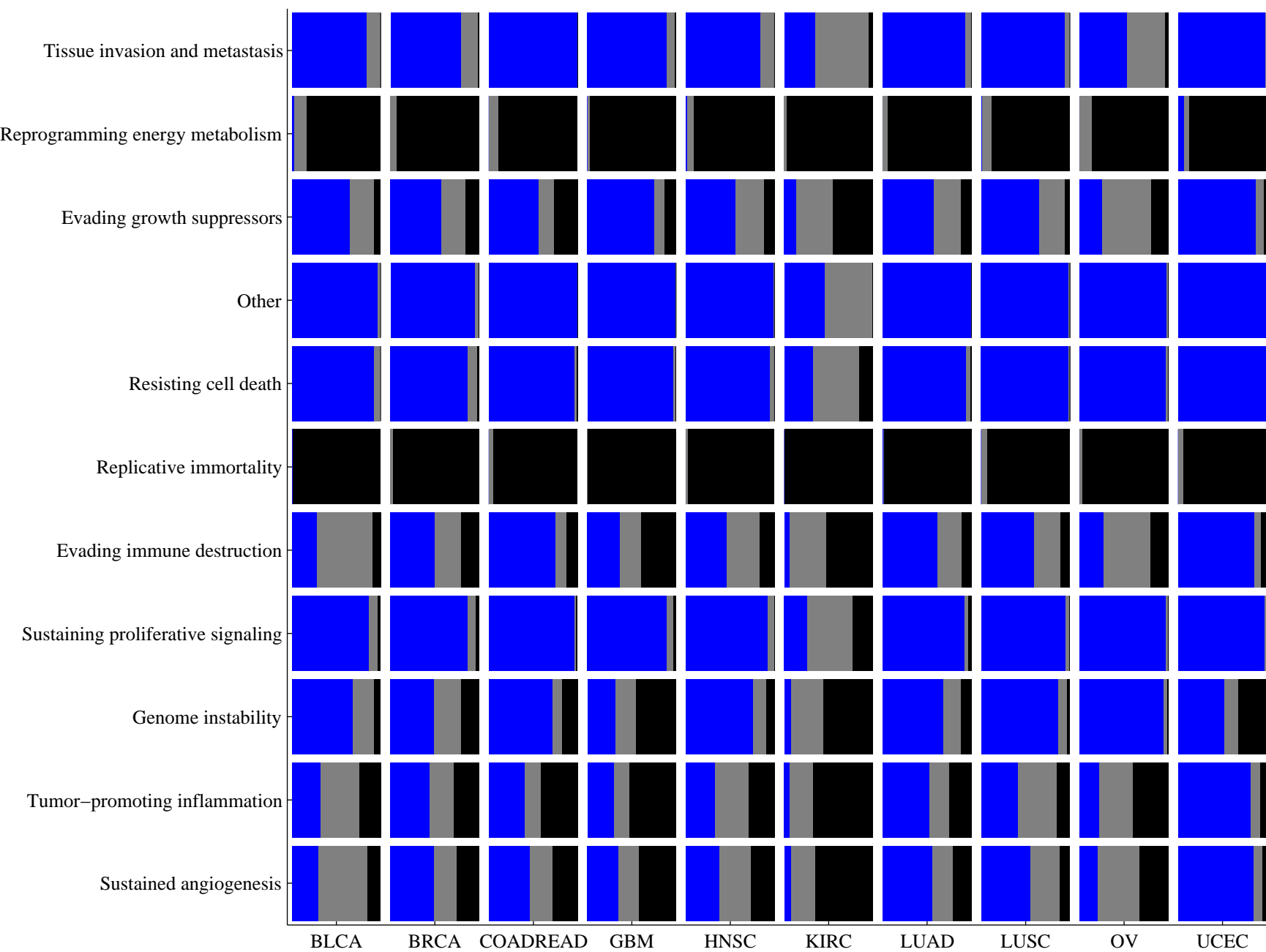

Supplement: Additional file 9: — Figure S7. Mutational investment in hallmarks explained by significantly mutated genes and non-significantly mutated genes. For each combination of a cancer type and a hallmark, blue areas indicate the percentage of samples that have a mutation in at least one gene that is linked to the hallmark and significantly frequently mutated according to Mutational Significance in Cancer (MuSiC) and/or Genomic Identification of Significant Targets In Cancer (GISTIC); grey areas indicate the percentage of samples that have a mutation in at least one gene that is linked to the hallmark, but none of these samples have a mutation in a significantly frequently mutated gene; black areas indicate the percentage of samples that have no mutated genes that link to the hallmark. BLCA, bladder urothelial carcinoma, BRCA, breast invasive carcinoma; CORE, colon/rectum adenocarcinoma; GBM, glioblastoma multiforme; HNSC, head and neck squamous cell carcinoma; KIRC, kidney renal clear cell carcinoma; LUAD, lung adenocarcinoma; LUSC, lung squamous cell carcinoma; OV, ovarian serous cystadenocarcinoma; UCEC, uterine corpus endometrioid carcinoma. [file 40880_2015_50_MOESM9_ESM.pdf]
